# Effect of postbiotic-toothpaste on salivary levels of IgA in 6- to 12-year-old children: Study protocol for a randomized triple-blind placebo-controlled trial

**DOI:** 10.3389/fped.2022.1042973

**Published:** 2022-12-12

**Authors:** Leila Basir, Eskandar Moghimipour, Afrooz Saadatzadeh, Bahman Cheraghian, Samaneh Khanehmasjedi

**Affiliations:** ^1^ Department of Pediatric Dentistry, School of Dentistry, Ahvaz Jundishapur University of Medical Sciences, Ahvaz, Iran; ^2^ Department of Pharmaceutics, School of Pharmacy, Nanotechnology Research Center, Ahvaz Jundishapur University of Medical Sciences, Ahvaz, Iran; ^3^ Department of Food and Drug Control, Faculty of Pharmacy, Ahvaz Jundishapur University of Medical Sciences, Ahvaz, Iran; ^4^ Department of Biostatistics and Epidemiology, School of Public Health, Alimentary Tract Research Center, Clinical Sciences Research Institute, Ahvaz Jundishapur University of Medical Sciences, Ahvaz, Iran; ^5^ Infectious and Tropical Diseases Research Center, Health Research Institute, Ahvaz Jundishapur University of Medical Sciences, Ahvaz, Iran

**Keywords:** probiotics, oral health, immunity, proteins, serum globulins, hydrogen-Ion concentration, saliva

## Abstract

**Background:**

Children in mixed dentition are highly at risk for dental caries, which is a major health issue worldwide. Despite their effect in controlling dental caries, using probiotics can be challenging. Therefore, it has been advised to use their inanimate forms, called postbiotics. We hypothesize that postbiotics can enhance the oral immunity.

**Methods:**

The aim of this triple-blind, randomized, placebo-controlled trial is to investigate the effect of postbiotic-toothpaste (*Bifidobacterium animalis subsp. animalis*) on salivary levels of Immunoglobulin A (IgA) and pH in children. Using comparing two means formula to calculate the sample size, for this trial 80 healthy 6- to 12-year-old children during mixed dentition with no cavitated dental caries will be selected by convenience sampling method and randomly allocated to two groups, postbiotic-toothpaste or placebo-toothpaste. Saliva samples will be gathered at baseline and four weeks after the intervention. The level of salivary IgA will be determined by ELISA and salivary pH will be measured using a pH meter. Data will be compared within and between groups using independent t-test and paired t-test, in case of normality, with a *p *< 0.05 as statistically significant.

**Discussion:**

If postbiotics-toothpaste prove to be effective in improving the oral immunity, they can be used to prevent dental caries and other oral diseases. The result of this study can help researchers who are working on the immunomodulatory effects of postbiotics in children.

**Trial registration number:**

Iranian Registry of Clinical Trials (IRCT), IRCT20191016045128N2. Registered on 7 March 2022.

## Background

### Background and rationale

Dental caries, the most prevalent oral disease, is still a major health issue worldwide (1). This multifactorial dynamic disease results in mineral loss of dental structure, is dental-biofilm-mediated, and can be modulated by diet (2). In children, it can affect their mastication function, alongside their smile and manner of speaking, and can be a significant blow to their oral health-related quality of life (3).

Children in mixed dentition, which usually begins at six years of age with the eruption of the first permanent tooth and continues until the exfoliation of the last primary tooth at around 12 years of age, are highly at risk for dental caries (4). This is mainly because teeth are most prone to caries in the first 2–4 years of eruption (5). Moreover, erupting teeth are out of alignment with their adjacent teeth and the gingiva in mixed dentition is sensitive and tender, making dental hygiene a challenge for children and their parents (4).

Contrary to the common belief that the rate of dental caries has declined in industrialized countries, this disease is still highly prevalent in these countries (6). It is believed that in European countries, 61% of 6- to 12-year-old children have experienced dental caries (7). A systematic review recently pointed out that the prevalence of dental caries has increased over the past few years in countries from the Middle East and North Africa region (6). So, it is evident that there is a great need for preventive interventions to decrease caries prevalence in children worldwide.

A rather novel preventive method is the usage of probiotics, which seems to be quite effective in controlling dental caries (8). Probiotics are “live microorganisms that confer a health benefit on the host when administered in adequate amounts” (9). Throughout the years, several studies have evaluated the effects of probiotics on oral health and stated that probiotics can reduce the numbers of pathogenic microorganisms, replace them (10, 11), and prevent or treat oral candidiasis, periodontitis, gingivitis, and halitosis (12, 13). Additionally, previous studies indicated the capability of probiotics to enhance the oral immune system and increase salivary Immunoglobulin A (IgA) secretion (14, 15).

Salivary IgA is an antimicrobial protein that is the first immune defense against *Streptococcus mutans*, the primary cariogenic bacteria. It inhibits bacterial adherence and reduces their colonization (16). A recent systematic review showed that, salivary IgA can have protective effects against dental caries (17).

Withal, it has recently been brought to attention that despite the aforementioned health benefits of probiotics, their usage can be quite challenging. Probiotics viability, storage stability, unfavorable effects in immunocompromised patients, antibiotic resistance, and transmission potential are a few of the issues (18, 19). To confront the side effects of probiotics, the usage of their inanimate form, also known as postbiotics, is increasingly gaining interest. According to the recent consensus statement of the International Scientific Association of Probiotics and Prebiotics (ISAPP), postbiotics are “preparation of inanimate microorganisms and/or their components that confers a health benefit on the host” (20). They have been shown to have an inhibitory influence on biofilm formation and alveolar bone loss (19), as well as antibacterial, antiviral, and antioxidant activities (18). Few studies, however, have evaluated the efficacy of postbiotics in enhancing oral immunity.

All in all, there seems to be a lack of enough data to fully support the hypothesis that postbiotics can elevate the salivary IgA concentration. In this protocol, a four-week triple-blind, placebo-controlled, randomized trial, investigating the effects of postbiotics on salivary IgA concentration in 6- to 12-year-old children is reported in detail.

### Objectives

In a study of 80 children, we aim to investigate if daily usage of postbiotic-toothpaste for 30 days can affect salivary levels of IgA in healthy 6- to 12-year-old children. Our secondary aim is to examine the impact of the intervention on salivary pH levels of the children. We hypothesize that daily usage of the postbiotic-toothpaste can elevate the salivary IgA concentration and improve oral immunity.

### Trial design

**Figure 1:**
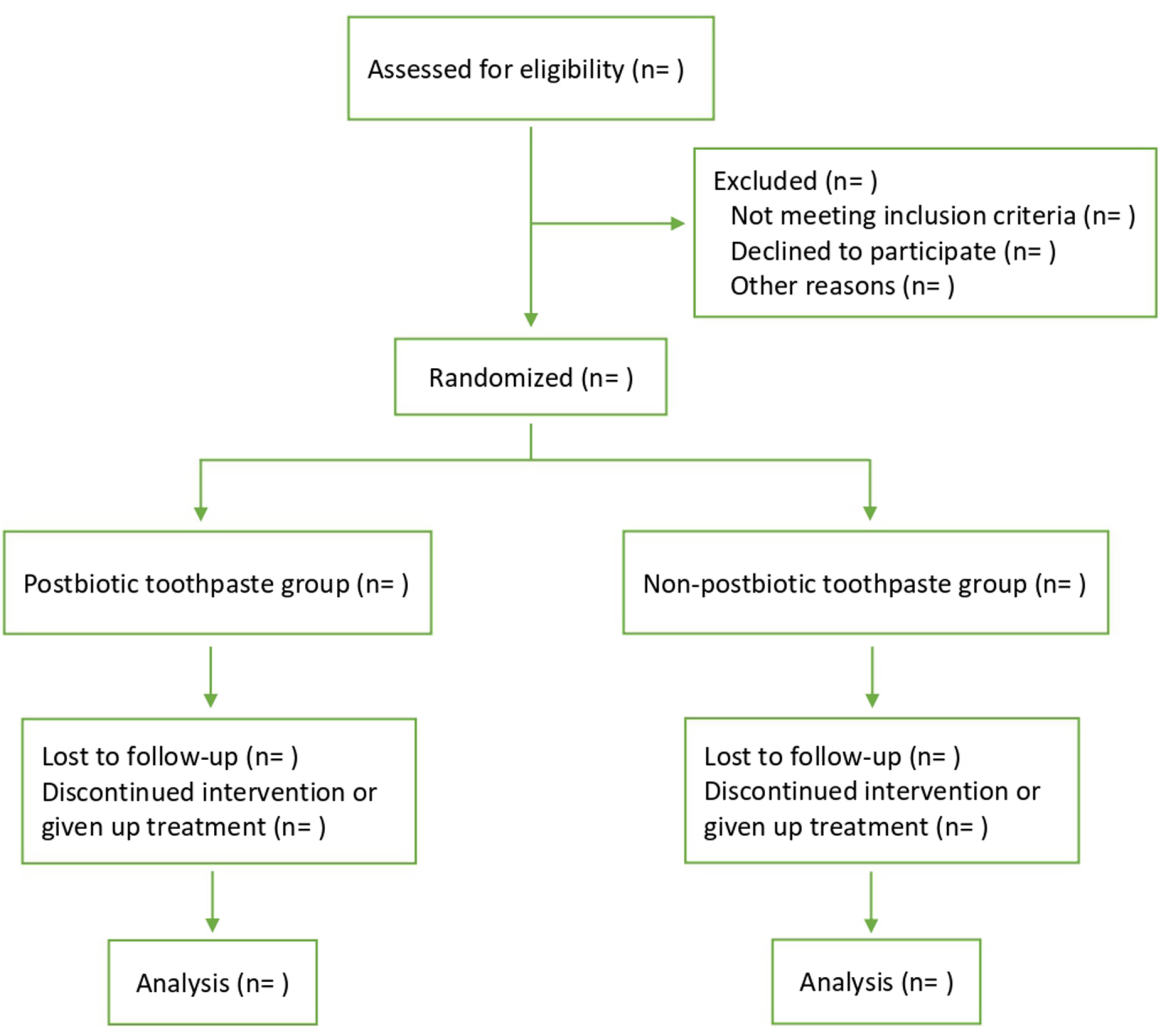
Study design flow diagram.

This will be a four-week, single-center, institutional-based, triple-blind, parallel, placebo-controlled, randomized clinical trial with a convenience sampling technique. Figure 1 shows the flow diagram of the study design from screening the patients to follow-up. This protocol is written according to the SPIRIT 2013 statement (21).

## Methods

### Study setting

The outpatients presenting to the Department of Pediatric Dentistry, School of Dentistry, Ahvaz Jundishapur University of Medical Sciences, Ahvaz, Iran will be recruited.

### Eligibility criteria

The inclusion criteria will be as followed: healthy, 6- to 12-year-old children, during the mixed dentition, no active or untreated carious lesions, no severe crowding, no marked inflammation in oral cavity.

The exclusion criteria will be the presence of any inflammatory disease and use of any medications, supplements, or probiotic intake within the last two weeks or during the intervention. The patients who fail to follow-up will also be excluded from the study.

### Study product and intervention

The postbiotic strain *Bifidobacterium animalis subsp. animalis* PTCC1631 will be used. The intervention will be in form of a postbiotic-toothpaste (*B. animalis subsp. animalis, 10*^*9*^^*CFU*^/*g* (22)). The postbiotic strain was ordered from the Persian Type Culture Collection center, a subset of the Iranian Research Organization for Science and Technology. The postbiotic and placebo-toothpaste are similar in packaging, color, taste, and scent, and the only difference is the absence of the postbiotic ingredient in the placebo-toothpaste. Table 1 shows the ingredients of the toothpastes.

**Table 1:** List of the ingredients of each toothpaste.

	Postbiotic-toothpaste (100 gr)	Placebo-toothpaste (100 gr)
*B. animalis subsp. animalis*	1–5 gr	
Sorbitol	30–40 gr	30–40 gr
Glycerin	20–30 gr	20–30 gr
Calcium carbonate	15–25	15–25
Carboxy methyl cellulose	1–2 gr	1–2 gr
Sodium saccharin	0.1–0.5 gr	0.1–0.5 gr
Sodium lauryl ether sulfate	2–5 gr	2–5 gr
Strawberry flavor	1–2 gr	1–2 gr
Distilled water	qs to 21 ml	qs to 21 ml

Eligible participants will be randomly allocated to two groups, intervention and control. The postbiotic-toothpaste will be given to the children in the intervention group and the children in the control group will receive the placebo-toothpaste. Children in both groups will be asked to brush their teeth twice a day (in the morning and at night) for two minutes, under the supervision of their parents, for four weeks. To eliminate any possible confounding effect of the method of tooth-brushing on the results, children and their parents will be instructed on how, when, and how long to brush their teeth at the beginning of the study.

In order to ensure participants adherence, parents will receive a message on a weekly basis to remind them the importance of tooth-brushing twice a day. Also, to motivate and encourage the children, they will be asked to record daily short videos of themselves while brushing their teeth and put it all together at the end of the four weeks.

### Outcomes

The primary outcome of this study will be the salivary IgA levels of the children and will be reported in mg/100ml. The secondary outcome will be the pH levels of saliva in children. The primary and secondary outcomes will be assessed at the baseline, before any intervention, and after four weeks of intervention.

### Sample size

To calculate the sample size, we used the following comparing two means formula (α=0.05, significance; $\beta \;=\;0.1;{\bar{x}}_{1}\;=\;1.4,\;{\bar{x}}_{2}\;\;=\;\;-3.1,S_{1}\;\;=\;\;4.1,\;\text{and}\;S_{2}\;\;=\;\;5.9$) based on the available literature (23) with maximum 30% falls during the follow-up, the final sample size was 40 participants per arm, 80 in total.


n=(Z(1−β)+Z(1−α/α22))2(S12+S22)(x¯1−x¯2)2

The participants will be selected using the convenience non probability sampling method. So, from the beginning of the study, any patient who meets the inclusion criteria and does not have the exclusion criteria will be selected and this process will continue until the sample size is reached.

### Randomization and blinding

To limit the potential confounding effect of the age of the children, they will be randomly allocated to two groups. Participants will be randomized according to the method of block randomization in blocks of six using a computer-generated random number sequence. This process will be done by an independent statistician who will also give the sealed envelope of the treatment codes to the pharmacist in charge of the production of toothpastes. Each of the eligible patients accepted into the study will be assigned a number from a consecutive range. This number will then be sent to the pharmacist.

During the study, the participants, the outcome assessor, and the data analyst will be blinded and will not have access to the nature of the toothpaste the subjects will be testing. The product will be divided into opaque sealed bags and the labels will not show the difference between the postbiotic and the placebo toothpastes. In case of a serious and adverse event, the blind will be broken and the pharmacist will have access to the sealed envelopes.

### Data collection

**Figure 2:**
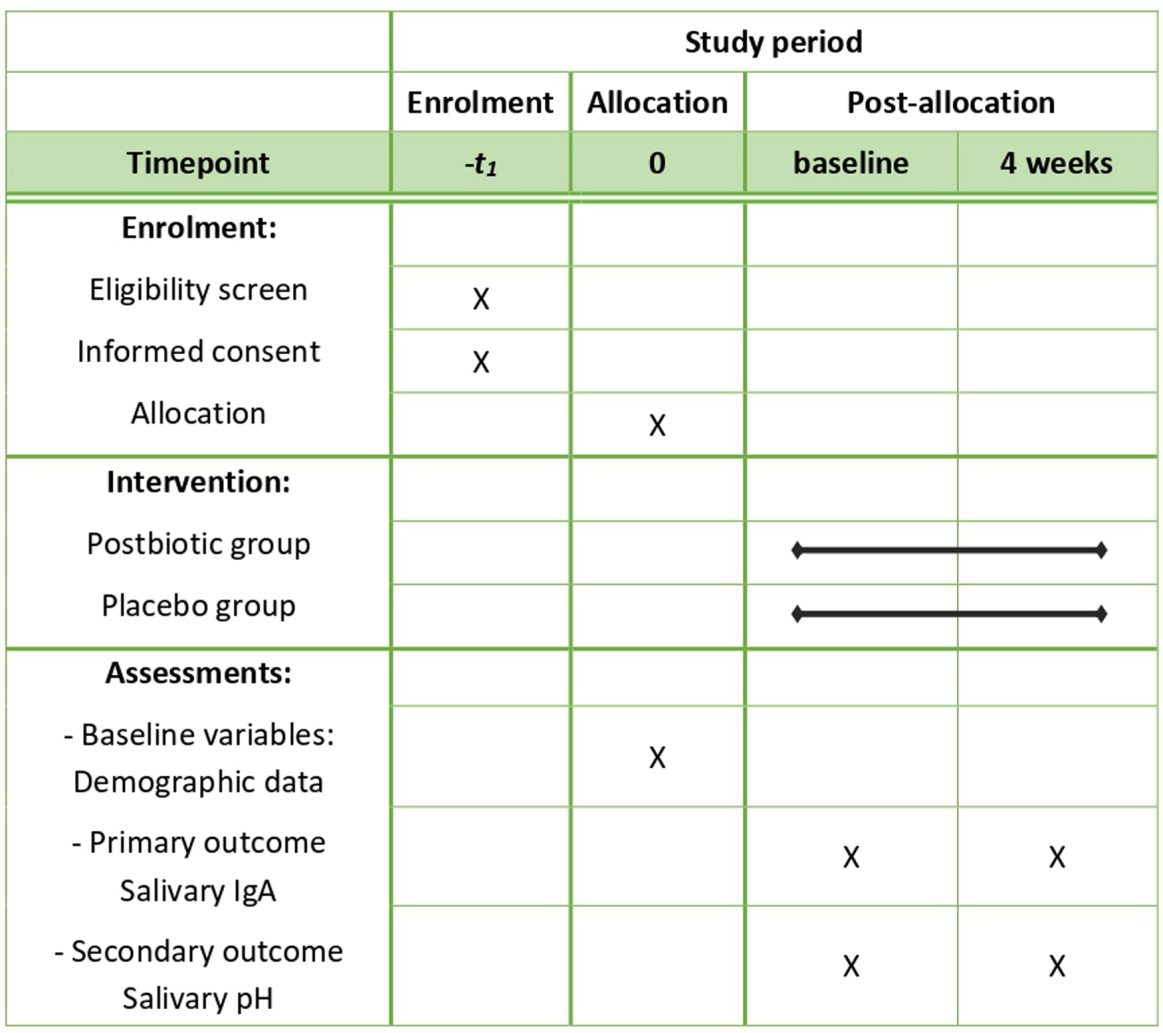
Schedule of enrolment, intervention, and assessments. IgA, Immunoglobin A.

At the beginning of the study, demographic information (age and gender), medical history, accompanying medication, and any usage of antibiotics and probiotics will be recorded. The eligible subjects will be told not to eat or drink anything for one hour before the saliva collection. Afterwards, one ml of the unstimulated whole saliva of children will be collected into sterilized plastic test tubes using the spitting method between 9.00 and 12.00 a.m. under controlled temperature (23°C). The saliva samples will be kept at −80°C until they are sent to laboratory, where the levels of salivary IgA will be determined with enzyme-linked immunosorbent assay (ELISA) previously described in detail by Sonesson et al. (24). The 96 well-plates will be coated overnight by anti-human *α*-chain-specific IgA. The next day, the wells will be washed with Phosphate-Buffered Saline (PBS) containing 0.05% Tween-20 and blocked by 1% Bovine Serum Albumin (BSA) at 37°C for 1 h. Diluted saliva samples and standards will be incubated in wells for 1 h at 37°C. The wells will be washed and biotinylated anti-human IgA antibody will be added. After washing away unbound biotinylated antibody, HRP-conjugated streptavidin will be pipetted to the wells. The wells will again be washed, a TMB substrate solution will be added to the wells and color develops in proportion to the amount of IgA bound. The Stop Solution will change the color from blue to yellow, and the intensity of the color will be measured at 450 nm. The results will be plotted against a standard curve and multiplied with the dilution factor. Immediately after sample collection in order to prevent degradation of the saliva, pH analysis will be carried out using portable pH meter (Hanna Instruments®, HI98191, RI, United States) which will be calibrated with distilled water for each time of use. At the follow-up session, outcomes will be reevaluated accordingly. A trained pediatric dentist will collect all the data. Figure 2 presents schedule of enrolment, intervention, and assessments.

### Statistical analysis

The analysis of the data will be based on the intention-to-treat analysis and all recruited participants will be considered. Data normality will be examined using the Kolmogorov-Smirnov test. The variables will be reported as mean ± SD or median. The percentage changes for each variable will be calculated using the following formula in which *E* and *B* stand for the end value and the baseline value of the variable, respectively.


$$\left[\;\frac{E-B}{B\;\times \;100}\;\right]$$

In case of meeting the assumption of normality, the significant changes between the two groups will be evaluated using the independent t-test, otherwise, Mann-Whitney analysis will be used. Comparison of the mean of variables at baseline with that of post-intervention within each group will be conducted using paired *t*-test or Wilcoxon signed-rank test. In order to control any possible confounding variable, the analysis of covariance (ANCOVA) will be used when necessary.

All analyses will be performed using the Statistical Package for Social Sciences, version 22.0 (SPSS, Inc., Chicago, IL, United States) with statistical significance at *p *< 0.05.

### Monitoring

To maintain the accuracy and quality of the trial, and to check its compliance with the protocol, it will be monitored regularly by research associates. The findings of the trial monitoring will be reviewed by the Data Monitoring Committee (DMC).

### Adverse events

Since the study product will be in form of a toothpaste, minimal harm is expected. However, an adverse event (AE) has been defined as any unfavorable discomfort in the mouth during the study period. All the children and their parents will be advised to report any AE immediately and in case of AE, participant(s) will be excluded from the study.

## Discussion

Existing literature indicates that postbiotics have advantageous effects on the human body. They have been proven to be effective in preventing diarrhea, acute gastroenteritis, pharyngitis, and laryngitis (24), and reducing respiratory tract infection in children (25). Numerous *in vitro* and *in vivo* studies have also shown the immunomodulatory effects of postbiotics, such as enhancing anti-inflammatory cytokines production (26), inhibiting tumor necrosis factor (TNF-*α*) secretion (27), and promoting T helper one immune response (28). In addition, probiotics and fermented foods supplementation significantly increased salivary IgA secretion rate (15). There have been few studies, however, that evaluated the influence of postbiotics on the secretion of salivary IgA.

Hishiki et al. in 2020 evaluated the effect of a heat-killed probiotic strain on viral infection of the respiratory tract in 3- to 6-year-old children and stated that after four months of intervention, the levels of secretory IgA in saliva were higher in the postbiotic group than that of the placebo group. Although, the results were not significant (29).

In another study in 2021, Lin et al. compared the effect of viable and heat-killed probiotic strains on oral immunity and found that after four weeks of viable and heat-killed probiotic administration, salivary IgA levels significantly increased in both groups (14).

In 2022, Lin et al. evaluated the effects of heat-killed probiotics and postbiotics on oral health and concluded that after four weeks of intervention, the levels of salivary IgA increased in both groups, and postbiotic supplementation can improve oral immunity (30).

Considering the shortage of clinical studies on the administration of postbiotics to increase oral immunity in children, through this randomized controlled trial, we propose to inspect the effect of postbiotics on children's oral immunity. If postbiotics-toothpaste prove to be effective in enhancing the oral immunity of children, they can be used as a new prophylactic method to prevent dental caries and other oral diseases. We expect the result of this study to be of great help to researchers working on the immunomodulatory effects of postbiotics in children.
